# Modulation of de novo purine biosynthesis leads to activation of AMPK and results in improved glucose handling and insulin sensitivity

**DOI:** 10.1186/2251-6581-13-51

**Published:** 2014-04-24

**Authors:** Satish Kumar Sadasivan, Balamuralikrishna Vasamsetti, Jaideep Singh, Nethra Siddaraju, Khaiser Mehdi Khan, Anup Mammen Oommen, Madanalli R Jagannath, Raghavendra Pralhada Rao

**Affiliations:** 1 Connexios life sciences private limited, JP nagara 3rd phase, Bangalore 560078, India

**Keywords:** Purines, AMPK, ADSL, T2DM, Obesity, Metabolic syndrome

## Abstract

**Background:**

AMP activated protein kinase (AMPK) regulates key metabolic reactions and plays a major role in glucose homeostasis. Activating the AMPK is considered as one of the potential therapeutic strategies in treating type-2 diabetes. However, targeting AMPK by small molecule mediated approach can be challenging owing to diverse isoforms of the enzyme and their varied combination in different tissues. In the current study we employ a novel strategy of achieving AMPK activation through increasing the levels of cellular AMP (an allosteric activator of AMPK) levels by activating the enzyme involved in AMP biosynthesis namely Adenylosuccinate lyase (ADSL).

**Methods:**

Rat primary hepatocytes were cultured under metabolic overload conditions (500 μM palmitate) to induce insulin resistance. ADSL was overexpressed in these hepatocytes and its effect on hepatic glucose output, and triglyceride accumulation was checked. In addition to this, ADSL was overexpressed in high fat diet induced obese mice by hydrodynamic tail vein injection and its effect on fasting glucose, glucose tolerance and pyruvate tolerance were checked.

**Results:**

Rat primary hepatocytes when cultured under metabolic overload conditions developed insulin resistance as measured in terms of failure of insulin to suppress the glucose output. Overexpressing the ADSL in these hepatocytes resulted in increased AMPK phosporylation and improved the insulin sensitivity and also resulted in reduced triglyceride accumulation and inflammatory cytokine levels. In addition to this, when ADSL was overexpressed in high fat diet induced obese mice, it resulted in reduced the fasting hyperglycemia (20% reduction), and increased glucose and pyruvate tolerance.

**Conclusions:**

This study indicates that activating ADSL can be a potential mechanism to achieve the activation of AMPK in the cells. This leads to a novel idea of exploring the purine nucleotide metabolic pathway as a promising therapeutic target for diabetes and metabolic syndrome.

## Introduction

Metabolic disorders, more specifically Type 2 Diabetes, obesity, cardiovascular diseases that result from both environmental and genetic factors are considered to be some of the fastest growing public health problems globally. These conditions may be associated with reduced insulin action and impaired glucose and lipid metabolism. Several therapeutic options available today differ in their mode of action, efficacy to lower blood glucose and associated complications and in their side effect profiles. Since T2DM is a progressive disease worsening with time, the available drugs need to be used in varied associations [1] and there is a pressing need to develop new therapeutic strategies to prevent and treat T2DM.

AMP-activated protein kinase (AMPK) is a phylogenetically conserved serine/threonine protein kinase, which acts as an energy sensing enzyme in the cells and is activated in response to cellular stresses that deplete the ATP levels in the cells [2,3]. It is shown to act as a central ‘fuel gauge’, and recent studies demonstrate AMPK as one of the probable targets for antidiabetic action of leading drugs like metformin [4,5] and thiazolidinediones [4,6]. In addition, chemical activation of AMPK with AICAR is shown to improve blood glucose and lipid profile. Taken together the above findings support the idea that AMPK can be an attractive therapeutic target for T2DM and insulin resistance [1,7]. However, developing a small molecule which regulates AMPK activity can be quite challenging considering the subtype complexity and tissue distribution of these isoforms [8].

The cellular energy status and AMPK activation are coupled by AMP, an allosteric activator of AMPK. Hence an indirect strategy to affect the AMPK activity would be to target the pathways that generate AMP in the cell. In the cell system, AMP is produced during purine nucleotide cycling reaction and the enzyme that produces AMP during biosynthesis is Adenylosuccinate lyase [(ADSL); EC 4.3.2.2.]. ADSL acts in two pathways of purine nucleotide metabolism. It catalyses the conversion of succinylaminoimidazole carboxamide ribotide (SAICAR) into aminoimidazole carboxamide ribotide (AICAR) in the purine *de novo* synthesis pathway, and it also catalyses the formation of adenosine monophosphate (AMP) from adenylosuccinate (S-AMP) in the purine nucleotide cycle [9,10]. We hypothesized that increasing the levels of ADSL in the cell would result in an increased AMP levels which in turn should lead to activation of AMPK and a consequent benefit on glycemic control. We evaluated this hypothesis using rat primary hepatocytes and high fat diet induced obese mouse model. Here we report that the ADSL overexpression in these two systems results in an increased insulin sensitivity, reduced hepatic glucose output and improved glucose handling.

## Methods

### Materials

An expression vector in which reading frame of ADSL is placed under the control of CMV promoter was obtained from Origene. In this expression vector ADSL is presented as un-tagged protein in order to avoid artefacts arising due to N or C terminal tags. Restriction enzymes with respective buffers and T4DNA ligase were from NEB. Trans-IT *invivo* gene delivery solution was from mirus biosciences. C57B6J mice were from Jackson laboratories USA.

### Cell culture experiments

Routinely, the freshly isolated rat primary hepatocytes were cultured in growth medium containing DMEM supplemented with 10% fetal calf serum in a humidified CO2 incubator at 37°C. For experiments involving ADSL overeexpression, primary hepatocytes were transfected with the expression plasmid using Lipofectamine™ 2000 (Invitrogen). 24 hours following the transfection, the cells were cultured in DMEM alone (referred to as normal culture condition) or in the DMEM medium containing 500 μM palmitic acid (referred to as Metabolic overload condition) for 24 hours. For experiments involving the measurement of glucose release, the adherent hpeatocytes were washed with phosphate buffered saline (PBS) and were incubated in the substrate medium (1% fetal calf serum, 5 mM lactate, 5 mM pyruvate, 100 IU penicillin, 100ug/ml streptomycin without glucose) with or without 30nM insulin for 6 hours. The glucose released into the media was estimated using glucose GOD - FS kit (days) following manufacturer’s instructions. HepG2 cells were cultured in DMEM supplemented with 10% fetal calf serum and a similar procedure described for rat primary hepatocytes was used for transfection. For estimation of the total TAG (triacyl glycerol) in the cells, HepG2 cells were cultured with or without palmiatate overlaod and after 48 hours cells were lysed in lysis buffer containing 50 mM Tris HCl (pH-8), 150 mM NaCl and 0.5% Triton-X. The clear lysaste was used for TAG estimation.

### Preparation of palmitate

Sodium palmitate (Sigma Aldrich) was dissolved at 500 mM concentration in 50% ethanol solution. This was further diluted to 5 mM using 10% FFA free BSA solution. This solution was filter sterilized and was used as stock solution.

### Estimation of ATP and AMP

Cells were washed in ice-cold PBS and the entire contents in the 24well plate were extracted into 95 μl of 0.3 M Perchloric acid and quickly transferred to centrifuge tube (1.5 ml). Contents were neutralized by adding 95ul of 2 M potassium hydroxide and the tube was vortexed and then centrifuged at 10,0000 rpm for 10 minutes at 4°C to remove acid-insoluble fractions. Supernatant was used for ATP and AMP estimations by LC-MS. RP-18 column was used for ESI-MS and (90% Acetonitrile +10% Water + 0.1% Ammonium formate) and 0.1% aqueous ammonium formate were used as mobile phase and gradient elution method was used. The levels of AMP and ATP was calculated based on the AUC obtained for the standard concentrations of the respective species.

### Western blot

Primary hepatocytes were lysed in lysis buffer (50 mM Tris HCl pH-8, 150 mM NaCl and 0.5% Triton-X) and the proteins in the lysate were separated by SDS-PAGE. The bands were transferred to nitrocellulose membrane and the blot was probed with phoshpo specific anti-AMPK antibody (phsopho AMPK-α Thr-172 rabbit monoclonal antibody reactive to rat isoform obtained from cell signaling) followed by appropriate secondary antibody. The bands were detected by chemiluminiscence.

### Gene expression analysis

Trizol extraction was used for RNA isolation from cells and tissues and the RNA was converted to cDNA. The expression levels of the gene were quantified by quantitative real time PCR using MesaGreen (SYBR green master mix) and gene specific primers. β-actin was used as endogenous gene control. The sequence of the primers used in this study is as follows:

MCP1 forward (rat) – 5′ATGCAGTTAATGCCCCACTC 3′

MCP1 reverse (rat) –5′TTCCTTATTGGGGTCAGCAC 3′

IL1- β forward (rat) – 5′TGACCCATGTGAGCTGAAAG 3′

IL1- β reverse (rat) – 5′GGGATTTTGTCGTTGCTTGT 3′

### Animals and groups

C57B6/J male mice were housed in group of 3–4 animals in standard polypropylene cages with stainless steel top grill having facilities for pelleted food and purified drinking water in bottle. Animals had access to food and water *ad libitum*. All animal experiments were approved by the Connexios Institutional Animal Ethics Committee (IAEC) which are in accordance with the ARRIVE guidelines and approved by the Committee for the Purpose of Control and Supervision of Experiments on Animals. Animals were housed at 22 ± 3°C, with a relative humidity of 50-70% on a 12 hour light and 12 hour dark cycle with artificial fluorescent tubes. 6 week old mice were fed on high fat diet (D12492 60% Kcal, fat from lard from Research Diets, US) for 13 weeks. After 13 weeks, the mice on high fat diet were evaluated for development of diabetic phenotype. Fasting glucose, body weight and oral glucose tolerance were considered for randomization. During randomization into groups, care was taken to make sure that body weight, fasting glucose and oral glucose tolerance values are not significantly different across these groups. Based on these parameters these mice were divided into 2 groups. One group of mice served as control (HFD control) and the other group served as ‘test group’ animals (designated as HFD + ADSL). Age and sex matched mice, which were kept on a normal chow diet (10% k.cal fat) were used as lean control in all the experiments.

### Gene delivery to mouse liver

The plasmid DAN used in these studies was endotoxin free. Each mouse received 20 μg of the plasmid DNA delivered through hydrodynamic tail vein injection as explained previously in literature [11,12]. Briefly, the plasmid DNA was suspended in gene delivery solution, the mouse was restrained and the solution was delivered through tail vein with a constant speed within 8 seconds. The volume of the gene delivery solution was 1/10th of the body weight of the mice and did not exceed 2.7 ml in any case. Mice in the control group (HFD control) received empty vector in which the ADSL sequences were absent. For the test group animals, the mice were injected with the plasmid expression vector. The mice received gene delivery once in 15 days. The lean control animals did not receive any plasmid DNA injections.

### Measurement of blood glucose levels

Blood was withdrawn from the tail tip and blood glucose was measured using Acuucheck instrument (Roche diagnostics). For the oral glucose tolerance test, animals were fasted for 6 hours, and blood glucose levels were measured at various time points following an oral glucose load of 2 g/kg (0, 15, 30, 60, 90, 120 minutes).

### Pyruvate tolerance test

Animals were fasted for 12 hours, and blood glucose levels were measured (0 minute glucose). After recording the 0 minute levels, a pyruvate load of 2 g/kg bodyweight were given to mice through oral gavage. Blood glucose levels were measured at time intervals of 15 minutes, 30 minutes, 60 minutes, 90 minutes, 120 minutes.

## Results

### ADSL expression is decreased in hepatocytes under disease mimicking conditions

**Figure 1:**
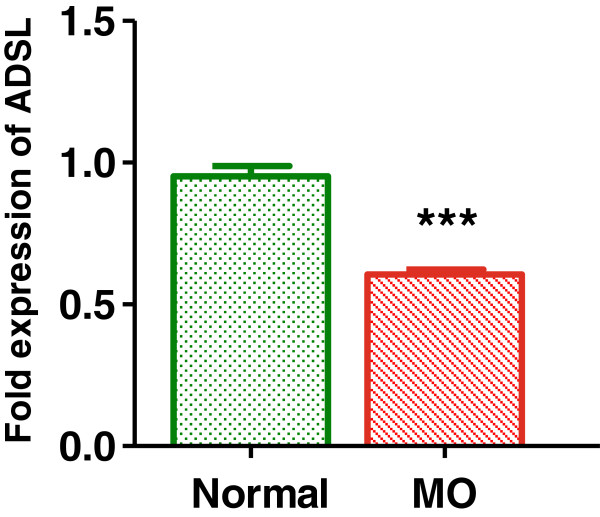
**ADSL expression in the rat primary hepatocytes.** Rat primary hepatocytes were cultured under normal or metabolic overload (MO) conditions and the level of expression of ADSL was checked by real time quantitative PCR. Beta actin was used as endogenous control and the level of expression of ADSL under normal condition was normalized to the value of 1. Unpaired students t-test was used to calculate the statistical significance *** (p < 0.001).

Earlier studies indicate that culturing hepatocytes with saturated fatty acid and pro-inflammatory agents can result in ‘pathophysiological condition’ *in vitro* wherein the hepatocytes show increased triglyceride accumulation [13], gluconeogenesis [14,15]– the hall marks of diabetic pathology. When rat primary hepatocytes were cultured in presence of 500 μM palmitate, we observed decreased insulin sensitivity, and increased inflammatory cytokine expression and increased TAG accumulation in HepG2 cells (discussed in subsequent sections) indicating that indeed palmitate overload in the culture media induced disease mimicking conditions. We used this system to represent ‘disease mimicking conditions’ *in vitro* and tested if the ADSL expression was different when cultured under palmitate overload conditions. As indicated in Figure 1, the expression levels of ADSL were significantly lower under metabolic overload conditions, indicating a possible role of ADSL for the observed pathology *invitro*.

### ADSL overexpression in the hepatocytes results in increase in AMP levels

**Figure 2:**
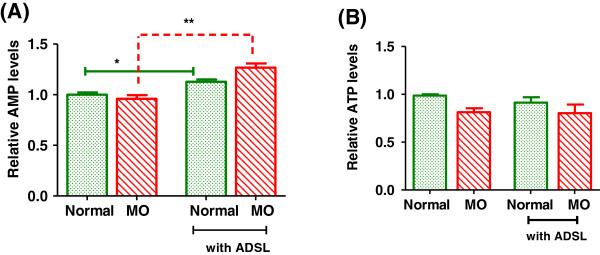
**AMP and ATP levels in the hepatocytes.** Rat primary hepatocytes were cultured under normal or metabolic overload (MO) conditions with or without ADSL overexpression. At the end of 24 hours, the AMP **(A)** and ATP levels **(B)** were checked LC-MS based measurements. Unpaired students t-test was used to calculate the statistical significance * (p < 0.05) ** (p < 0.01).

We overexpressed ADSL in rat primary hepatocytes using expression vector in which ADSL was cloned under the control of a strong CMV promoter. In order to confirm that the ADSL protein exhibits the expected activity in the cells, we checked for the levels of AMP, which is a product of enzymatic action of ADSL on its substrate IMP. As indicated in the Figure-2A, cells overexpressing ADSL exhibited increased AMP levels compared to respective controls. As expected, ADSL overexpression resulted in increased AMP levels in the hepatocytes [16]. With ADSL overexpression there was about 12% increase in the AMP levels in the hepatocytes cultured under normal conditions. However, under disease mimicking conditions ADSL overexpression resulted in about 25% increase in the AMP levels. In our experiments we saw that under metabolic overload conditions, the ATP levels were slightly reduced (Figure 2B) and notably the expression of ADSL did not alter the ATP levels either under normal or metabolic overload conditions (Figure 2B).

### ADSL overexpression increases AMPK phosphorylation in primary hepatocytes

**Figure 3:**
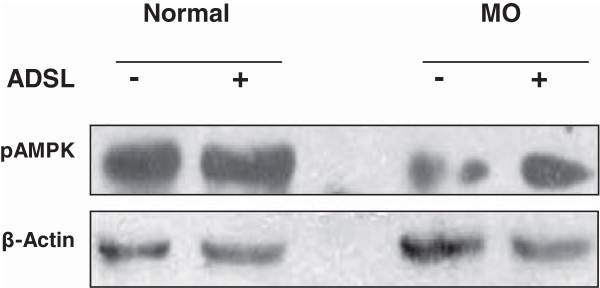
**Phospho-AMPK levels following ADSL overexpression.** Rat primary hepatocytes were cultured under normal or metabolic overload (MO) conditions with or without ADSL overexpression. At the end of 24 hours the cells were lysed and the pAMPK protein levels (Thr-172 phosphorylated) were visualized by western blotting.

AMPK is a major regulator of cellular energy homeostasis and its enzymatic activity is stimulated through phosphorylation of threonine-172 on the catalytic subunit alpha, by an upstream AMPK kinase and through allosteric activation by AMP through CBS (cystathionine b-synthase) domains on the gamma subunits. AMPK is typically susceptible to the AMP/ATP ratio, which in turn is influenced by cellular and environmental stress, such as heat shock, hypoxia, ischemia and metabolic poisoning activates AMPK. In our study, we found that subsequent to overexpression of ADSL, the AMP levels were increased while the ATP levels remained unchanged indicating an increase in AMP/ATP ratio (Figure-2A and B) following ADSL expression. This increase indeed reflected in an increase in the AMPK phosphorylation. AS shown in Figure 3, when ADSL was overexpressed under normal culture conditions, the increase in the AMPK levels was very marginal (lane2). However the AMPK phosphorylation showed significant increase with ADSL overexpression when the cells were cultured under metabolic overload conditions (Figure 3, lane4). This correlates well with the extent of increase in the AMP levels under corresponding conditions (Figure 2).

### ADSL overexpression restores insulin-mediated repression of glucose release in primary hepatocytes

**Figure 4:**
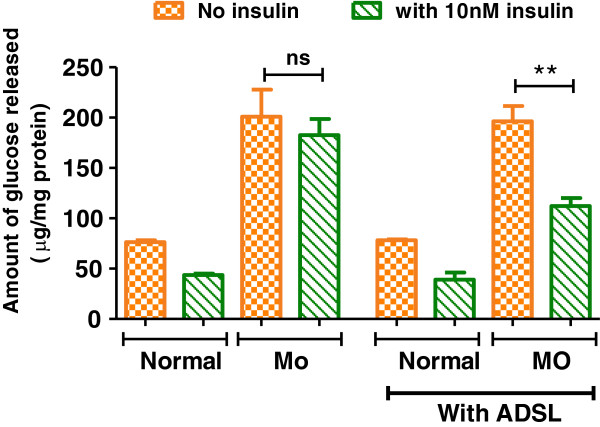
**Effect of ADSL overexpression on hepatic glucose output and insulin sensitivity.** Rat primary hepatocytes were cultured under normal Normal or metabolic overload (MO) conditions. At the end of 24 hours the hepatic glucose output was estimated by measuring the glucose released into the culture media either in the absence (control) or presence of 10nM of insulin (+insulin) as explained in materials and methods. Glucose released into the media was normalized to total cellular protein. Unpaired students t-test was used to calculate the statistical significance ** (p < 0.01).

Elevated hepatic glucose production is a major contributor towards increased fasting glucose levels in diabetes [1,17]. Insulin represses hepatic glucose output from the liver and this is dysregulated in diabetic conditions leading to elevated fasting glucose levels. As shown in Figure 4, hepatocytes cultured under normal conditions respond to insulin treatment and show about 43% decrease in the glucose output. However, when cultured under disease mimicking conditions (MO, 500 μM palmitate), the glucose output from hepatocytes was about 2.5 fold higher compared to normal and insulin treatment resulted only in about 10% decrease in the glucose output indicating the loss of insulin sensitivity. ADSL overexpression in this background could restore the insulin sensitivity - as shown in Figure 4, when ADSL was overexpressed in the cells cultured under disease mimicking conditions, they respond to insulin treatment and there was about 40% decrease in the glucose output in these cells.

### ADSL overexpression decreases TG accumulation in the liver

**Figure 5:**
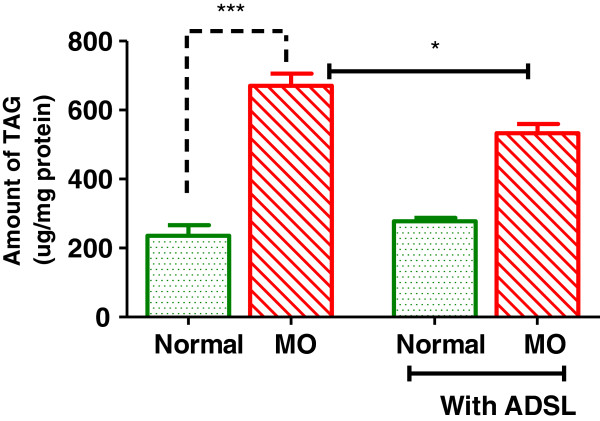
**Effect of ADSL overexpression on hepatic triglyceride content.** HepG2 cells were cultured under Normal or metabolic overload (MO) conditions. At the end of 48 hours, the cells were lysed and triglyceride (TAG) levels were measured. The TAG levels were normalized to total cellular protein. Unpaired students t-test was used to calculate the statistical significance ** (p < 0.01).

AMPK activation system is known to be a regulator of ectopic lipids metabolism [1]. Activation of AMPK in the liver is shown to decrease fatty acid biosynthesis and increase mitochondrial fatty acid oxidation [1], owing to this, AMPK activation is shown to significantly decrease triglyceride storage in ectopic tissues like liver [18]. We checked if ADSL overexpression and concomitant AMPK activation can have any impact on liver triglyceride levels. As shown in Figure 5 when hepatocytes were cultured under metabolic overload conditions, there was about 2.8 fold increase in the triglyceride levels. When ADSL was overexpressed in this background it resisted the increase in TG indicating the beneficial effect of ADSL activation under metabolic overload conditions.

### ADSL overexpression decreases expression of acute phase proteins in liver

**Figure 6:**
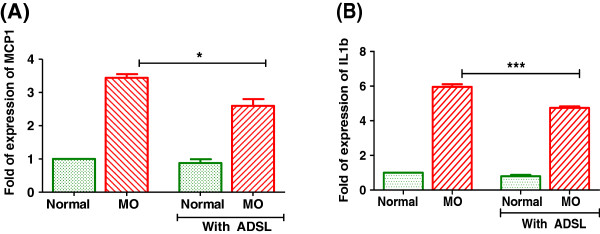
**Effect of ADSL overexpression on inflammatory cytokines.** Rat primary hepatocytes were cultured under Normal or metabolic overload (MO) conditions for 24 hours. The m-RNA levels of MCP-1 **(A)** or IL-1b **(B)** were checked by real time quantitative PCR. Beta actin was used as endogenous control. Unpaired students t-test was used to calculate the statistical significance * (p < 0.05), ***(p < 0.001).

Several prospective studies show that T2DM is a case of chronic low grade inflammation and acute phase proteins are shown to be elevated in diabetic conditions [19] and they are even proposed as predictive markers for development of diabetes [20]. When cultured with palmitate load, the expression levels of acute phase proteins were increased in hepatocytes (Figure 6). Expression of ADSL in this background resulted in significant reduction in the levels of acute phase proteins.

### ADSL expression in the mouse liver

**Figure 7:**
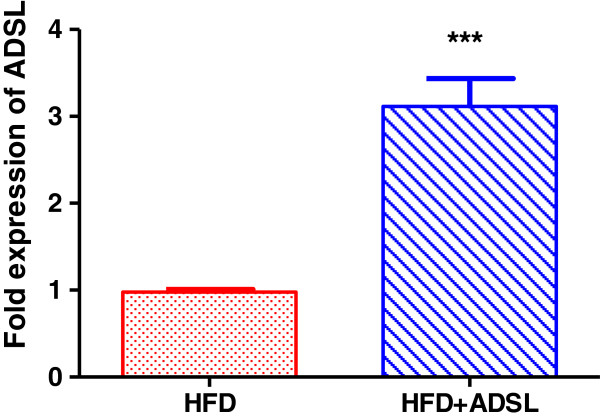
**ADSL overexpression in HFD mouse liver.** Mice were fed on high fat diet for 13 weeks and were divided into two groups (n = 8 in each group). For one group, DNA construct expressing the ADSL gene was injected through tail vein (HFD + ADSL), and control group received plasmid that does not express ADSL (HFD control). The gene delivery was conducted every. At the end of the study, (4 weeks later) RNA was isolated from liver and ADSL gene expression levels were quantified using Q-PCR. b-actin was used as house keeping gene control. Unpaired students t-test was used to calculate the statistical significance * (p < 0.05), ***(p < 0.001).

In order to further evaluate the effect of ADSL in vivo, we overexpressed ADSL in the liver in DIO mice. Mice which were kept on high fat diet for 13 weeks were delivered with ADSL expression vector through hydrodynamic gene delivery as explained in materials and methods. Gene delivery was conducted intermittently (every 15 days) in order to obtain a sustained overexpression. At the end of the study (4 weeks later), the mice were sacrificed and the expression of the ADSL in the gene delivered mice was confirmed by real time quantitative PCR and as shown in Figure 7, there was about 3 fold increase in the ADSL levels in the test group mice.

### ADSL expression improves fasting glucose and pyruvate tolerance

**Figure 8:**
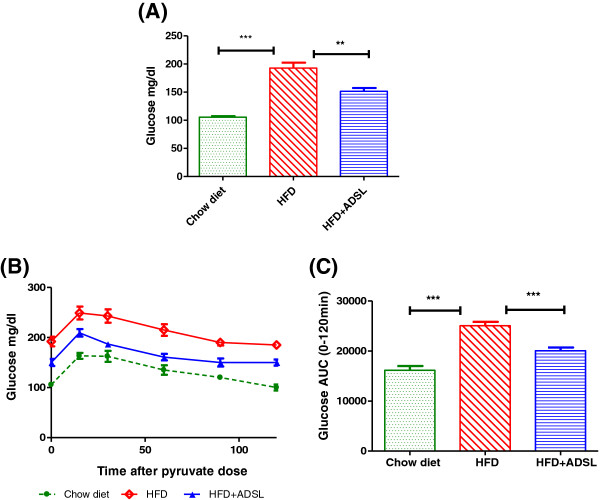
**Effect of ADSL overexpression on fasting glucose and pyruvate tolerance.** Mice were fed on high fat diet for 13 weeks and divided into two groups (n = 8 in each group). For one group, DNA construct expressing the ADSL gene was delivered to liver (HFD + ADSL), and control group received plasmid that does not express ADSL (HFD). 3 weeks later (post gene delivery), both groups of mice were fasted for 12 hours and fasting glucose levels were recorded **(A)** and pyruvate tolerance was measured as indicated in materials and methods **(B)** and the area under the curve during the pyruvate tolerance test was calculated **(C)**. Unpaired students t-test was used to calculate the statistical significance ** (p < 0.01), ***(p < 0.001).

3 weeks following the gene delivery, the fasting glucose levels were measured in the mice. As indicated in Figure 8A mice expressing ADSL exhibited about a 20% decrease in the fasting glucose levels. High fat diet induced mice exhibit increased glucose output following challenge with pyruvate a gluconeogenic substrate, and this is due to uncontrolled gluconeogenesis. The pyruvate tolerance test is used routinely as a measure of gluconeogenesis index invivo. When challenged with pyruvate, the HFD control mice showed increased glucose production and there was 56% increase in the glucose-AUC when compared to mice fed on chow diet (Figure 8B and 8C) indicating unregulated gluconeogenesis in these mice. When ADSL was overexpressed, the glucose output in response to pyruvate challenge was significantly low compared to HFD control mice - when compared to untreated HFD mice, the ADSL expressing mice exhibited 20% decrease in the glucose output as measured by glucose – AUC (Figure 8C).

### ADSL expression improves glucose tolerance

**Figure 9:**
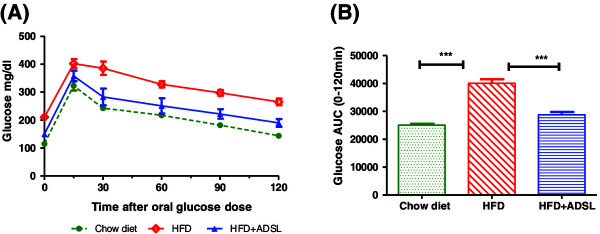
**Effect of ADSL overexpression on glucose tolerance.** Mice were fed on high fat diet for 13 weeks and divided into two groups (n = 8 in each group). For one group, DNA construct expressing the ADSL gene was delivered to liver (HFD + ADSL), and control group received plasmid that does not express ADSL (HFD). 3 weeks later mice were fasted for 6 hours and glucose tolerance was measured as indicated in materials and methods. **(A)** – Glucose levels at different time points after oral glucose load, **(B)** – Area under the curve between 0 min to 120 min.

As indicated in Figure 9A, mice on HFD background show increased glucose levels at all time points following an oral glucose load. In terms of glucose AUC, the HFD mice show about 60% increase in the glucose output compared to lean mice on chow diet (Figure 9B). overexpressing the ADSL in HFD mice resulted in marked improvement in the glucose tolerance and in terms of glucose AUC, the ADSL overexpressing mice showed about 28% decrease in the glucose output indicating a significant effect of ADSL on glucose utilization *in vivo*. In this study the ADSL was overexpressed only in the liver, but not in muscle and adipose indicating that improved glucose uptake can be attributed to improved insulin sensitivity of the liver. The relative importance of liver as compared to other peripheral tissues like muscle and adipose tissue in the uptake of an oral glucose load has been estimated in earlier studies and these studies have demonstrated that approximately 10% of the oral glucose load is trapped by the liver on first passage through the portal system [21]. Also, the liver takes up approximately 50% of the total amount of glucose escaping initial hepatic uptake and thus is a major contributor of the glucose uptake [22,23]. Insulin increases the levels of glucokinase [24,25] and glycogen synthase activity in the liver [26] which finally results in trapping the glucose inside the liver as glycogen and this reaction in effect can facilitate the glucose uptake by GLUT2 a glucose concentration dependent glucose transporter present on the liver cells. Hence insulin sensitivity acts as a positive influence on hepatic glucose uptake, although GLUT2 is an insulin independent transporter. This is further evidenced by studies involving human subjects [27].

## Discussion and conclusions

Purine nucleotides, apart from being a part of nucleic acids, act as key regulators of vital functions of cell including cell metabolism, cell proliferation and cell death. They are the essential carriers of chemical energy such as ATP. Purine metabolite state modulates the AMP/ATP ratio and can impact mitochondrial function as well as AMPK activity affecting key cellular functions such as skeletal muscle oxidative capacity and glucose oxidation, hepatic glucose output, glucose sensitivity and islet insulin secretion – all of which can affect glucose homeostasis. Under diabetic condition, metabolic stress can lead to reduced mitochondrial function which can impact the cellular ATP pools. This in conjunction with reduced glucose metabolism due to insulin resistance, can result in reduced purine metabolites, making it rate limiting for further cellular processes. Earlier studies indicate that under insulin resistance and diabetic conditions, the mitochondrial function is compromised leading to reduced ATP synthesis [28,29].

The importance of purine metabolism in metabolic syndrome is supported further by literature evidences. Adenosine A1 receptor signaling is shown to be important for insulin controlled glucose homeostasis in mice [30] and mice with adenosine receptor (A1AR) deletion is shown to exhibit age dependent fat accumulation in the body [31]. Adenosine, is a metabolic byproduct of ATP utilization, is shown to have a positive effect on insulin sensitivity and glucose handling [32,33]. Despite several reports on the importance of the purinergic signaling in glucose metabolism, the direct effect of manipulating the purine metabolism on glucose homeostasis is lacking in the literature.

ADSL is a homotetramer [34] involved in the purine biosynthesis pathway. Several studies conducted in the past focus on the significance of ADSL in neurophysiology – mutations leading to ADSL deficiency results in various neurological disorders ranging from mild mental retardation in some cases to fatal neonatal encephalopathy with hypokinesia [35-37]. However, evaluation of ADSL from metabolic syndrome perspective is novel. Our present study demonstrates that by increasing the AMP levels under disease conditions, one can get the benefits on glycemic parameters possibly through AMPK activation. Although in the present study we did not extensively validate the small molecule targetability and safety profile of ADSL with small molecule, it is interesting to note that overexpression of ADSL in the mouse model did not have any detectable pathology, and did not show any noticeable change in the behavior/health during the course of our study. The overexpression strategy used in this study has limitations and one cannot control the extent of activation. Using a small molecule based approach can provide a lot more flexibility in terms of extent of activation. Hence, with a small molecule based approach one can have control over the extent of physiological effect. Our study provides preliminary data supporting the possibility of exploring ADSL as a target for metabolic syndrome.

## Abbreviations

ADSL: Adenylosuccinate lyase; AMP: Adenosine 5′ monophosphate; AMPK-AMP: activated protein kinase; ATP: Adenosine triphosphate; AUC: Area under the curve; HFD: High fat diet; MO: Metabolic overload; PBS: Phosphate buffered saline; TAG: Triacyl glycerol.

## Competing interests

All the authors listed in the manuscript conducted the work exclusively at wet lab and animal facility of connexios life sciences and all the authors were employees of connexios life sciences at the time when the research work was carried out. The authors declare they have no competing interests.

## Authors’ contribution

AO, RPR and MRJ were involved in the designing the study and data analysis. SKS, BV, KMK, NS and JS were involved in conducting the experiments. RPR & MRJ were involved in manuscript writing. All authors read and approved the final manuscript.
